# Reversal of Multidrug Resistance in Human Colon Cancer and Human Leukemia Cells by Three Plant Extracts and Their Major Secondary Metabolites

**DOI:** 10.3390/medicines5040123

**Published:** 2018-11-13

**Authors:** Jun-Xian Zhou, Michael Wink

**Affiliations:** Institute of Pharmacy and Molecular Biotechnology, Heidelberg University, Im Neuenheimer Feld 364, 69120 Heidelberg, Germany; junxian.zhou@stud.uni-heidelberg.de

**Keywords:** multidrug resistance (MDR), plant extracts, plant secondary metabolite (PSM), ABC transporter

## Abstract

**Background:** We studied the effect of three plant extracts (*Glycyrrhiza glabra*, *Paeonia lactiflora*, *Eriobotrya japonica*) and six of their major secondary metabolites (glycyrrhizic acid, 18β glycyrrhetinic acid, liquiritigenin, isoliquiritigenin, paeoniflorin, ursolic acid) on the multidrug resistant human colon cancer cell line Caco-2 and human leukemia cell line CEM/ADR 5000 as compared to the corresponding sensitive cell line CCRF-CEM, and human colon cancer cells HCT-116, which do not over-express ATP-binding cassette (ABC) transporters. **Methods:** The cytotoxicity of single substances in sensitive and resistant cells was investigated by MTT assay. We also applied combinations of extracts or single compounds with the chemotherapeutic agent doxorubicin or doxorubicin plus the saponin digitonin. The intracellular retention of the ABC transporter substrates rhodamine 123 and calcein was examined by flow cytometry to explore the effect of the substances on the activity of ABC transporters P-glycoprotein and MRP1. Real-time PCR was applied to analyse the gene expression changes of ABCB1, ABCC1, caspase 3, caspase 8, AhR, CYP1A1, and GSTP1 in resistant cells under the treatment of the substances. **Results:** All the substances moderately inhibited cell growth in sensitive and resistant cells to some degree. Whereas ursolic acid showed IC_50_ of 14 and 22 µM in CEM/ADR 5000 and Caco-2 cells, respectively, glycyrrhizic acid and paeoniflorin were inactive with IC_50_ values above 400 μM. Except for liquiritigenin and isoliquiritigenin, all the other substances reversed MDR in CEM/ADR 5000 and Caco-2 cells to doxorubicin. Ue, ga, 18ga, and urs were powerful reversal agents. In CEM/ADR 5000 cells, high concentrations of all the substances, except *Paeonia lactiflora* extract, increased calcein or rhodamine 123 retention in a dose-dependent manner. In Caco-2 cells, all the substances, except liquiritigenin, retained rhodamine 123 in a dose-dependent manner. We also examined the effect of the plant secondary metabolite (PSM) panel on the expression of ABCB1, ABCC1, caspase 3, caspase 8, AhR, CYP1A1, and GSTP1 genes in MDR cells. **Conclusions:** The extracts and individual PSM could reverse MDR in CEM/ADR 5000 and Caco-2 cells, which overexpress ABC transporters, in two- and three-drug combinations. Most of the PSM also inhibited the activity of ABC transporters to some degree, albeit at high concentrations. Ue, ga, 18ga, and urs were identified as potential multidrug resistance (MDR) modulator candidates, which need to be characterized and validated in further studies.

## 1. Introduction

Cancer cells are often found to develop resistance to anticancer drugs, not only to a single or a group of drugs, but also to many other structurally and functionally unrelated drugs, even if the cells have never been exposed to the other drugs. This is called multidrug resistance (MDR), which is a key reason of chemotherapy failure in various cancers [1,2]. Three major mechanisms have been implicated in MDR: (1) decreased uptake of transporter-dependent hydrophilic drugs into cells; (2) various cellular changes that reduce drug toxicity and promote cell survival opportunities, for example, altered by drug metabolism, and decreased apoptosis; and (3) an increased drug efflux, mediated mainly by ATP-binding cassette (ABC) transporters [3]. Recent studies suggested some new mechanisms for MDR, for example, cell-cell interactions, mechanotransduction, lysosomal drug sequestration, and so on [4,5].

Export of hydrophobic drugs by ABC transporters across the cell membrane, using the energy of ATP binding, reduces the total intracellular drug concentration, or these transporters redirect the cytotoxic drugs away from target organelles [6]. P-glycoprotein (P-gp, MDR1 or ABCB1) and multidrug resistance-associated protein (MRP) were the earliest identified ABC transporters [7,8]. Their over-expression contributes to the MDR phenotype of cancer cells [9]. Cancer cells can develop resistance towards apoptosis, the execution of which requires caspases (caspase 3, 8, 9, etc.), and this capability—evading apoptosis—is a hallmark of cancer [10,11,12]. Besides reduced apoptosis, altered metabolism of drugs can also influence MDR. The aryl hydrocarbon receptor (AhR) is a transcription factor which regulates phase I (cytochrome P450, CYPs) and phase II metabolic enzyme (microsomal epoxide hydrolases, glutathione S-transferases (GSTs), sulfotransferases) expressions [13,14]. Many anticancer drugs undergo CYP-mediated metabolism and excretion and thus lose their efficacy [15]. One of the phase II metabolic enzyme GSTs, GSTpi, is involved in the conjugation reactions of drugs in many different tumors [16], and its expression level is often high in MDR cancer cells [17].

Chemotherapeutic agents such as doxorubicin often show a general high toxicity and thus limited efficacy [18]. Drug combinations may improve their efficacy, and even show synergistic effects [19]. The steroidal saponin digitonin and other saponins can increase the permeability of cell membranes [20] and may generate synergism in drug combinations. Some plant extracts and their secondary metabolites (PSM) can inhibit ABC transporter and reverse MDR [21]. Here, three plant extracts from Traditional Chinese Medicine (TCM), the root extracts of *Glycyrrhiza glabra*, *Paeonia lactiflora* (mixed with *Paeonia veitchii*), and the leaf extract of *Eriobotrya japonica* and six of their major secondary metabolites (glycyrrhizic acid, 18β glycyrrhetinic acid, liquiritigenin, and isoliquiritigenin from *G. glabra*, paeoniflorin from *P. lactiflora*, and ursolic acid from *E. japonica*) were investigated as potential substrates for ABC transporters and modulators of MDR.

*Glycyrrhiza glabra* (Fabaceae) root has been used in traditional medicine in Europe and Asia for thousands of years [22,23] to treat hepatitis C, inflammation, and other health conditions. It possesses anti-inflammatory, anticancer, antiviral, antimicrobial, antitussive, expectorant, and other biological activities [24,25]. *Paeonia lactiflora* (Paeoniaceae) is an ornamental garden plant and used in traditional medicine, especially in Traditional Chinese Medicine [23]. Depending on the processing of roots, two kinds of herbal medicines are on the market: white dried root without bark mainly originates from *Paeonia lactiflora*, while red dried root with bark originates from *P. lactiflora* or *P. veitchii* [26]. *Paeonia lactiflora* extract has anti-inflammatory, antiviral, anticancer, and antibacterial effects [27]. *Paeonia veitchii* was found to have activities against enterovirus infections [28]. *Eriobotrya japonica* (Rosaceae) is a traditional medicine plant used to treat cough and as an expectorant. It has anti-inflammatory and anti-diabetic properties [29].

**Figure 1:**
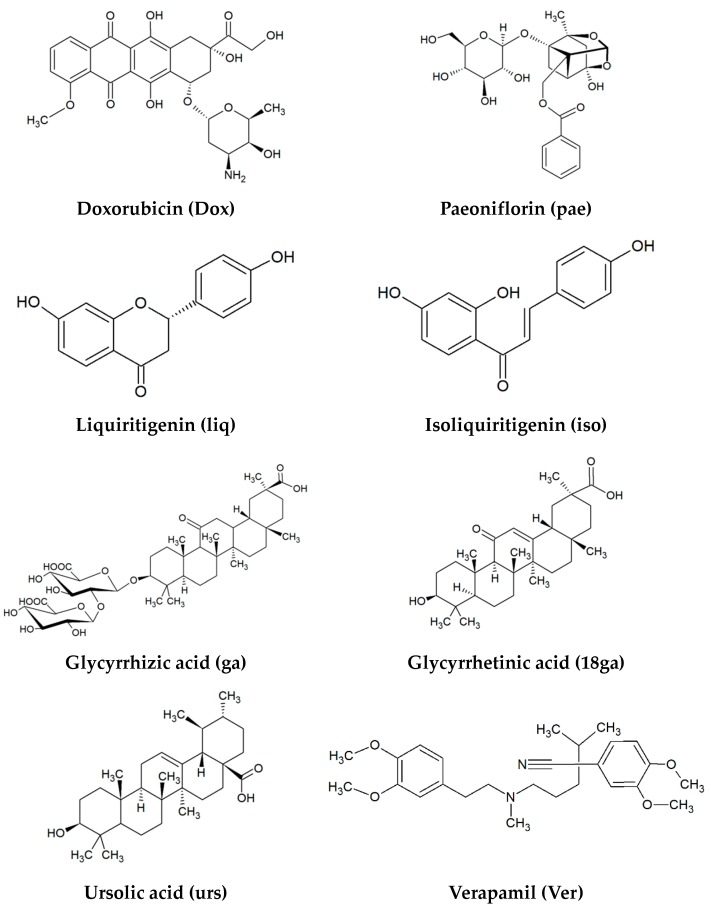

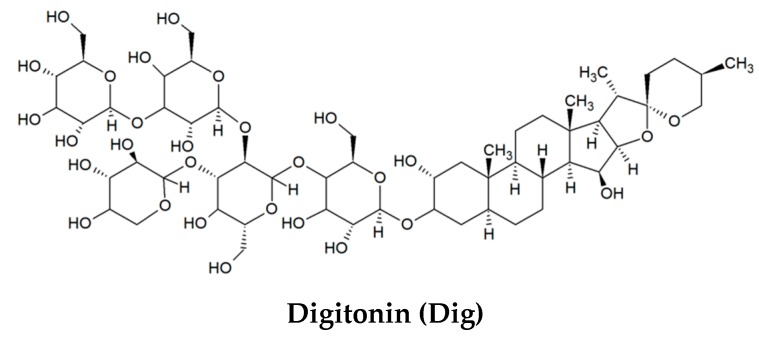
Chemical structures of compounds (and their abbreviations) used in this study.

The six main secondary metabolites from the three TCM plants possess various pharmacological activities. The triterpenoid saponin glycyrrhizic acid showed anti-cancer and anti-inflammatory activities [30,31,32]. 18β Glycyrrhetic acid is the aglycone of glycyrrhizic acid; it exhibits anti-malarial and anti-inflammatory effects, and excellent anticancer potential in some cancer cells [33,34,35]. Liquiritigenin, a major flavonoid of *G. glabra*, possesses antitumor, anti-inflammatory, and neuroprotective effects [36,37]. The flavonoid isoliquiritigenin has antiproliferative ability in cancer cells [38,39,40]. The content of the monoterpene paeoniflorin in *P. lactiflora* is above 1.6% [27]. Paeoniflorin exhibits anti-inflammatory, immunoregulatory, neuroprotective, and anti-cancer properties [41,42,43,44]. Ursolic acid, a pentacyclic triterpenoid in *E. japonica* and widely distributed in various plants [45], has a wide spectrum of pharmacological activities, for example, antimutagenic, and anti-cancer activities [46,47]. The structures of the compounds are shown in Figure 1.

We investigated the influence of the three plant extracts and six major PSM from them (Figure 1) on the MDR cancer cells CEM-ADR 5000 and Caco-2 as compared to the sensitive CCRF-CEM and HCT-116 cells and explored their possible mechanisms. Of special interest was the ability of the PSM panel to exert synergistic MDR reversal for doxorubicin in two- and three-drug combinations. We focused on the modulation of ABC transporters, apoptosis and drug metabolism. In this context, we analysed expression changes of ABCB1, ABCC1, caspase 3, caspase 8, AhR, CYP1A1, and GSTP1 genes in resistant cells after the treatment with plant extracts and secondary metabolites.

## 2. Materials and Methods

### 2.1. Materials

*Glycyrrhiza glabra* was purchased from Caesar & Lorentz GmbH (Hilden, Germany). *Paeonia lactiflora* was bought with *Paeonia veitchii* as a mixture in a pharmacy in China. *Eriobotrya japonica* was obtained from Kräuter Schulte (Gernsbach, Germany). Human T lymphoblast CCRF-CEM and leukaemia cell line CEM/ADR 5000 were kindly provided by Professor Dr. Thomas Efferth (Institute of Pharmacy and Biochemistry, University of Mainz, Mainz, Germany). Human colon cancer cells HCT-116 were obtained from Professor Dr. Stefan Wölfl (Institute of Pharmacy and Molecular Biotechnology, Heidelberg University, Heidelberg, Germany). Human epithelial colorectal adenocarcinoma cells Caco-2 were bought from the German Collection of Microorganisms and Cell Cultures (DSMZ, Braunschweig, Germany).

The 3-(4,5-Dimethylthiazol-2-yl)-2,5-diphenyltetrazolium bromide (MTT), doxorubicin, and verapamil were bought from Sigma-Aldrich (Darmstadt, Germany). Digitonin was from Carl Roth (Karlsruhe, Germany). Glycyrrhizic acid, 18β glycyrrhetinic acid, isoliquiritigenin, liquiritigenin, paeoniflorin, ursolic acid were obtained from Baoji Herbest Bio-Tech (Baoji, China).

### 2.2. Plant Extraction

The dried roots of *Glycyrrhiza glabra*, *Paeonia lactiflora*, and the dried leaves of *Eriobotrya japonica* were powdered and immediately extracted using ultrasound with 100% methanol (for *G. glabra*), 50% ethanol (for *P. lactiflora*), and 96% ethanol (for *E. japonica*), respectively, at room temperature for 50 min. After cooling down, the extracts were centrifuged at 4000 rpm for 12 min and filtered. The solvent was freeze dried and the dry extracts of *Glycyrrhiza glabra* (Ge), *Paeonia lactiflora* (Pe) and *Eriobotrya japonica* (Ue) were stored at 4 °C for use.

### 2.3. Cell Culture and Viability by MTT Assay

Suspension cells (CCRF-CEM and CEM/ADR 5000 cells) were cultured in RPMI 1640 media containing 10% FBS, 100 U/mL penicillin-streptomycin and 2 mM l-glutamine. Adherent cells (Caco-2 and HCT-116 cells) were cultured in DMEM containing 10% FBS, 100 U/mL penicillin-streptomycin and 2 mM l-glutamine. All cells were incubated at 37 °C with 5% CO_2_.

The MTT assay was modified from Mosmann [48]. For the adherent cells Caco-2 and HCT-116, cells with a density of 6 × 10^4^ were seeded in 96-well plates and incubated for 24 h at 37 °C. Media were removed and various doses of substances prepared in media were added to the plates and incubated for 24 h (for HCT-116 cells) or 48 h (Caco-2 cells). Then media were removed, and 0.5% MTT (dissolved in media) was added and further incubated at 37 °C for 2–4 h. Subsequently, the plates were centrifuged at 400 rpm for 10 min, and the absorption was read at 570 nm with the Tecan Nano Quant infinite M200 PRO Plate Reader (Tecan, Männedorf, Switzerland).

For the suspension cells CCRF-CEM and CEM/ADR 5000, cells with a density of 30 × 10^4^ were seeded in 96-well plates, and various doses of substances were added. The plates were incubated for 48 h, and MTT was added to a final concentration of 0.5% and further incubated for 2–4 h. The incubation was terminated, plates were centrifuged for 10 min, and the absorption was read at 570 nm using the Tecan Nano Quant infinite M200 PRO Plate Reader. Doxorubicin was used as a positive control.

### 2.4. Effect of Two- and Three-Drug Combinations on Cell Viability

A non-cytotoxic dose (below IC_30_) of a single plant extract or secondary metabolite was added together with different concentrations of the chemotherapeutic agent doxorubicin (two-drug combination) or doxorubicin plus 0.5 μM digitonin (three-drug combination). The MTT assay was used to test whether the test compounds can increase the sensitivity of CEM/ADR 5000 cells and Caco-2 cells to doxorubicin. We compared the IC_50_ of doxorubicin in drug combinations with the IC_50_ of doxorubicin applied alone.

### 2.5. ABC Transporter Assay with the Substrate Calcein-AM in CCRF-CEM and CEM/ADR5000 Cells

Calcein-acetoxymethyl ester (calcein AM) and rhodamine 123 (rho 123) are nontoxic, fluorescent dyes and substrates of P-gp and MRP1. By measuring their cellular fluorescence retention with flow cytometry, the activity of ABC transporters (P-gp and MRP1) under drug treatment in MDR cancer cells can be detected [49,50,51,52,53]. 

30,000–50,000 cells per well were seeded in a 24-well plate, and three concentrations of drugs (plant extracts and SM) (IC_50_ × 1, IC_50_ × 5, IC_50_ × 10) were added separately to the cells and incubated for 1.5 h at 37 °C. The cell suspension containing 1% DMSO without drugs was used as the control group. The P-gp and MRP1 substrate verapamil [54] (50 μM) was used as a positive control. After incubation with different compounds, the suspension cells were transferred into 2 mL Eppendorf tubes and separated from the media by centrifugation. The cells were washed twice with ice-cold PBS. Then 500 μL media containing calcein-AM (the final concentration of calcein-AM was 1.25 μM for CCRF-CEM and 2.5 μM for CEM/ADR 5000 cells) were added to each tube for 1.5 h incubation at 37 °C. Afterwards, the cells were separated from the media by centrifugation, and washed twice with ice-cold PBS. Then the cells were resuspended in ice-cold PBS and moved to FACS tubes. The cellular fluorescence intensity was measured with FACS at 525 nm.

### 2.6. ABC Transporter Assay with the Substrate Rhodamine 123 in CCRF-CEM, CEM/ADR 5000 Cells, and Caco-2 Cells

For CCRF-CEM cells, the procedure was the same as that with calcein-AM; only calcein-AM was replaced with 10 μM rhodamine 123 (rho 123). For CEM/ADR 5000 cells, 30,000–50,000 cells per well were seeded in a 24-well plate and pre-incubated at 37 °C for 1 h in the presence of 10 μM rho 123. Then the suspension cells were transferred into 2 mL Eppendorf tubes and separated from the media by centrifugation. The cells were washed twice with ice-cold PBS. Then 500 μL media containing various concentrations of drugs were added to each tube for 1.5 h incubation at 37 °C. 1% DMSO was used as the control group, and 50 μM verapamil was used as a positive control. Afterwards, the cells were separated from the media by centrifugation, and washed twice with ice-cold PBS. Then the cells were resuspended in ice-cold PBS and moved to FACS tubes. The cellular fluorescence intensity was determined by FACS at 525 nm.

For Caco-2, cells were grown in a 96-well plate until a monolayer was formed in each well. Fresh media containing different concentrations of drugs were added to the cells (each treatment was in triplicate). Verapamil was used as a positive control. The cells were incubated at 37 °C for 5 h. Afterwards, the media were removed and new media containing 10 μM rho 123 were added to the cells for 1.5 h further incubation. Then the media were removed and the cells were washed once with PBS. After addition of 100 μL PBS containing 0.1% Triton to each well the fluorescence was read at 535 nm with the Tecan Nano Quant infinite F200 PRO Plate Reader (Tecan, Männedorf, Switzerland).

### 2.7. Gene Expression Analysis by by Real-Time PCR

CEM-ADR 5000 and Caco-2 cells were treated with various single substances for 48 h. Then, RNA were extracted with the RNA purification kit (EURx, Roboklon, Berlin, Germany) following the protocol of the supplier. After the purification, RNA was reverse-transcribed to cDNA with FastGene Scriptase Basic cDNA Kit (NIPPON GENETICS, Dueren, Germany) following the protocols of the supplier. Then real-time PCR were performed with SensiFAST SYBR No-ROX kit (Bioline, Luckenwalde, Germany) or ORA™ qPCR Green ROX L Mix, 2X (HighQu, Kraichtal, Germany). In each reaction, the following agents were added: 0.1 μL DMSO, 0.1 μM forward and reverse primers (Table 1), 100–500 ng cDNA template, 5 μL SYBR qPCR Mix or Green ROX L Mix, and then add water to 10 μL final volume. Each reaction was conducted in triplicate. After centrifuging, the reaction plate was sealed with transparent qPCR folie and placed into Roche LightCycler^®^ 96 Real-Time PCR instrument (Roche, Penzberg, Germany). The reaction steps were set like this: 1 cycle of 95 °C—2 min initial denaturation, 40 cycles of 95 °C—5 s denaturation, 40 cycles of 60–65 °C 10 s annealing, 40 cycles of 72 °C 20 s extension, and 4 °C cooling. The data was analysed using 2^−ΔΔCt^ following Yuan et al. [55].

### 2.8. Reversal Ratio

The reversal ratio, also referred to as “fold-sensitization,” or “MDR ratio” etc., evaluates the reversal effect of a chemosensitizer by comparing the IC_50_ values of a toxic drug combined with a nontoxic concentration of a chemosensitizer and used alone [54].

Here the reversal ratio is calculated with the following equation:

$$\mathrm{Reversal}\;\mathrm{ration}=\frac{{\mathrm{IC}}_{50},\;\mathrm{dox}\;\mathrm{alone}}{{\mathrm{IC}}_{50},\;\mathrm{dox}\;\mathrm{in}\;\mathrm{combination}}$$

It suggests whether the combination of a substance with dox can reverse the resistance of a cell line to dox.

### 2.9. Combination Index (CI)

For the two- and three-drug combination in CEM/ADR 5000 and Caco-2 cells, combination index (CI) was calculated with the following equations according to Zhao et al. [56].

For two-drug combinations:

$$\mathrm{CI}=\frac{C_{dox,50}}{{\mathrm{IC}}_{50,dox}}+\frac{C_{SM}}{{\mathrm{IC}}_{50,SM}}$$

For three-drug combinations:

$$\mathrm{CI}=\frac{C_{dox,50}}{{\mathrm{IC}}_{50,dox}}+\frac{C_{SM}}{{\mathrm{IC}}_{50,SM}}+\frac{C_{dig}}{{\mathrm{IC}}_{50,dig}}$$

C*_dox_*_,50_ is the IC_50_ of dox in combination.
IC_50,*dox*_ is the IC_50_ of dox alone in a cell line.
C*_SM_* is the constant concentration of a single substance in combination.
IC_50,*SM*_ is the IC_50_ of a single substance (a secondary metabolite or a plant extract) in the cell line.
C*_dig_* is the concentration of digitonin used in combination. It is 0.5 μM in this study.
IC_50,*dig*_ is the IC_50_ of digitonin in the cell line.

The interpretation of CI was according to Chou [57]: CI < 0.1 very strong synergism (+++++), 0.1–0.3 strong synergism (++++), 0.3–0.7 synergism (+++), 0.7–0.85 moderate synergism (++), 0.85–0.9 slight synergism (+), 0.9–1.10 nearly additivity (±), 1.10–1.20 slight antagonism (-), 1.20–1.45 moderate antagonism (--), 1.45–3.3 antagonism (---), 3.3–10 strong antagonism (----), and >10 very strong antagonism (-----). NR = not relevant.

### 2.10. Statistical Analysis

All the results are expressed as the mean ± SD from at least three independent experiments. The data were analysed with SigmaPlot^®^ 11.0 (Systat Software, San Jose, CA, USA) and GraphPad Prism 6 (Graphpad Software, San Diego, CA, USA). Statistical significance was assessed with *t*-test. When *p* < 0.05, the difference was regarded as significant.

## 3. Results

### 3.1. Cytotoxicity of Single Substances against Drug Sensitive and Resistant Cells

The antiproliferative activity of the individual substances in CCRF-CEM, CEM/ADR 5000, HCT-116 and Caco-2 cells was measured using MTT assay (Table 2). CEM/ADR 5000 and Caco-2 cells overexpress ABC transporters and are therefore less sensitive to doxorubicin than CCRF-CEM or HCT-116, which do not express them. The cytotoxic effect of the plant substances can be considered as moderate. Digitonin and ursolic acid are the most active PSM in our panel and exhibit IC_50_ values between 0.7 and 20 µM. The IC_50_ values of ga and pae were above 400 μM, indicating that they are not toxic to cells (Table 2).

### 3.2. Combinations Enhance the Cytotoxicity of Doxorubicin

A nontoxic dose of a single extract or secondary metabolite was combined with different concentrations of doxorubicin or doxorubicin plus 0.5 μM digitonin to test whether the two- and three-drug combinations can restore or even increase the cytotoxicity of doxorubicin and overcome the drug resistance in CEM/ADR 5000 (Table 3) and Caco-2 cells (Table 4). In CEM/ADR 5000 cells, all two- and three-drug combinations lowered the IC_50_ of doxorubicin, and reversed the resistance of CEM/ADR 5000 to doxorubicin with a reversal ratio >1. The combination index (CI) varied and indicated interactions from synergism to antagonism. Three-drug combinations were even more effective than two-drug combinations, namely, digitonin enhanced the reversal effect of all single substances. In Caco-2 cells, all the substances showed a similar effect as in CEM/ADR 5000 cells, except liquiritigenin (liq) and isoliquiritigenin (iso). When doxorubicin was combined with liq or iso alone, the IC_50_ of doxorubicin was not lowered but increased, the reversal ratio was <1, and the CI indicated antagonism. Only when iso was used in three-drug combination, the IC_50_ of doxorubicin was somewhat reduced. Importantly, Ue, ga, 18ga, and urs were identified as powerful reversal agents.

### 3.3. Activity towards ABC Transporters in CEM/ADR 5000 Cells

**Figure 2:**
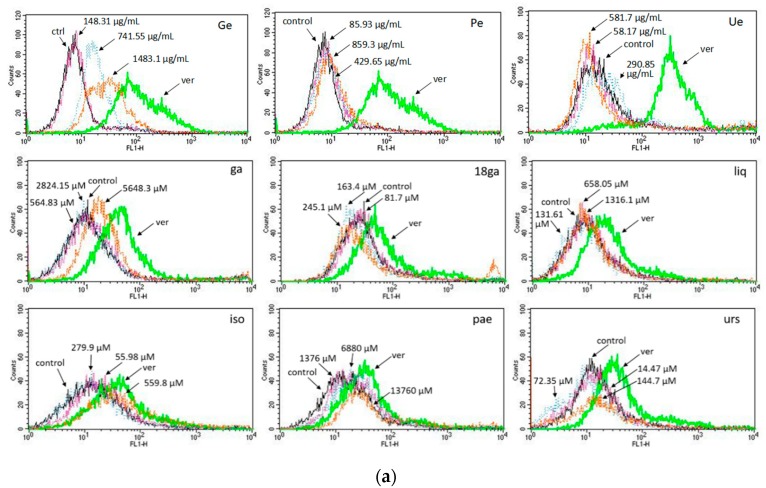

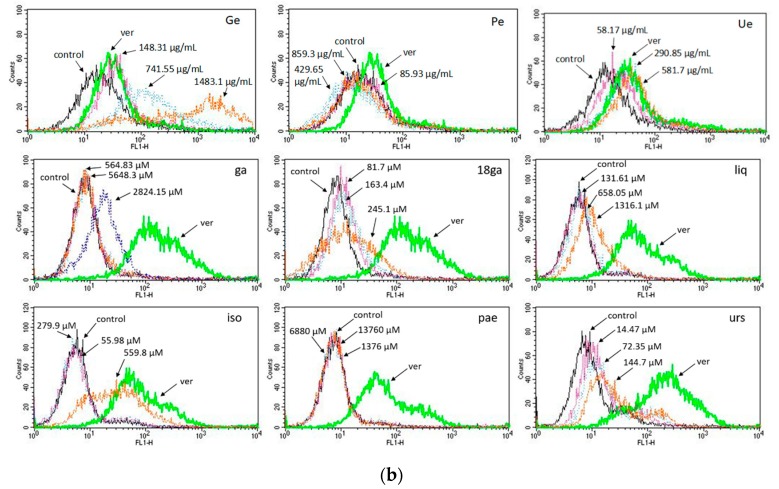
Histograms of flow cytometry of calcein-AM (**a**) and rhodamine 123 (**b**) retention in CEM/ADR 5000 cells after 1.5 h treatment with three concentrations (IC_50_, IC_50_ × 5, and IC_50_ × 10) of a single substance compared to the treatment with 50 μM verapamil (positive control). All experiments were repeated at least three times.

In a next set of experiments, we explored if the synergism observed in Table 3 and Table 4 might be explained by an inhibition of ABC transporters. Figure 2 shows the cellular calcein AM (Figure 2a) and rhodamine 123 (rho 123) (Figure 2b) retention in CEM/ADR 5000 cells after incubation with the test substances. 50 μM verapamil (ver) was used as a positive control. In calcein AM assay, high concentrations of Ge, Ue, ga, iso and pae promoted calcein retention, whereas Pe, Ue, 18ga, liq, and urs had no effect (Figure 2a). In rho 123 assay, Ge, Ue, ga, 18ga, liq, iso, and urs showed a dose-dependent rho 123 retention, although the effect was weaker than that of verapamil. We conclude, that some of our PSM do interfere with p-gp activity.

**Figure 3:**
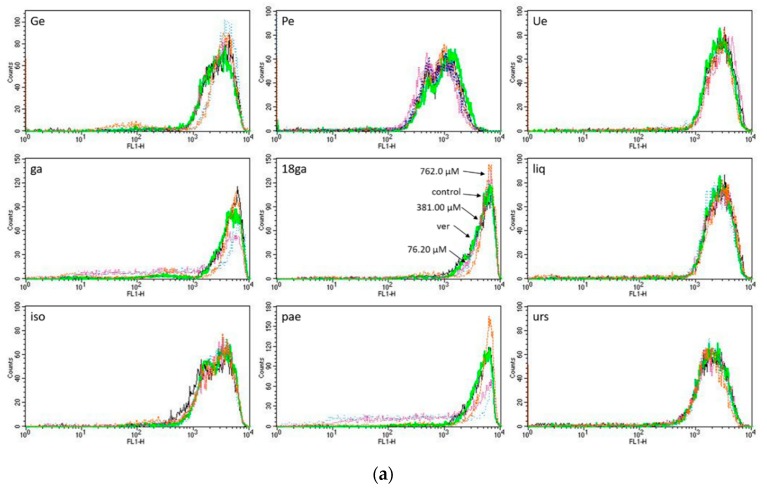

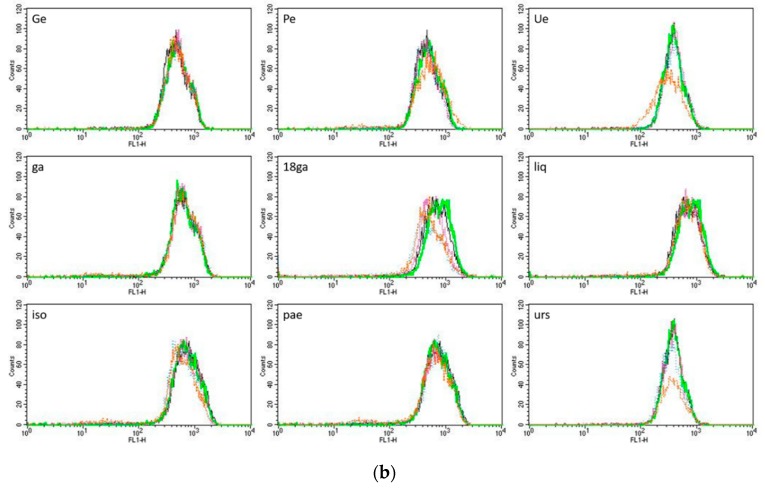
Histograms of flow cytometry of calcein-AM (**a**) and rhodamine 123 (**b**) retention in CCRF-CEM cells after treatment with three concentrations (IC_50_, IC_50_ × 5, and IC_50_ × 10) of single substance compared to the treatment with 50 μM verapamil (positive control).

In the sensitive CCRF-CEM cells, no ABC transporter-mediated drug efflux occurred, the fluorescence of calcein and rhodamine 123 in the control group, positive control verapamil group and drug treatment groups exhibited few or almost no differences, as shown in Figure 3a,b.

### 3.4. Rhodamine 123 Retention in Caco-2 Cells

**Figure 4:**
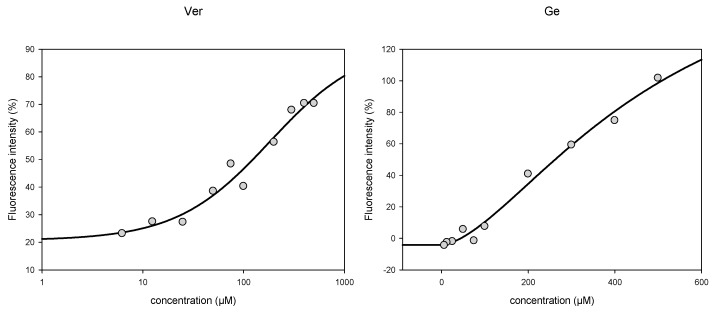

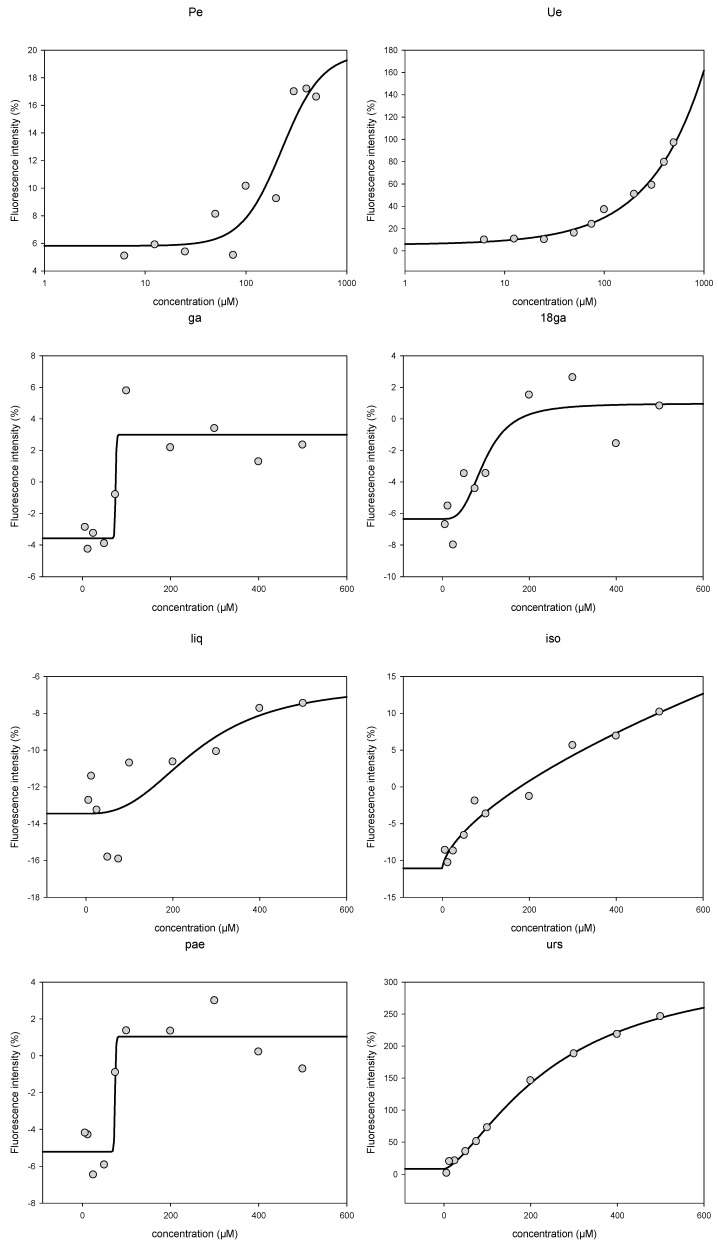
Fluorescence intensity of rhodamine 123 in Caco-2 cells after 5 h treatment with a single substance. Verapamil was used as a positive control. All data were from at least three experiments represented as the mean ± SD.

In analogy to the experiments with CEM/ADR 5000 cells, Caco-2 cells were treated with our set of single substances for 5 h, and then incubated with rho 123 for 1.5 h to determine the activity of p-gp. The cellular fluorescence intensity was recorded by spectrometry. As shown in Figure 4, the positive control verapamil, and all the substances except liq demonstrated a dose-dependent trend of rho 123 retention; the higher the concentration, the stronger the cellular fluorescence. Some substances such as ga, 18ga, iso, and pae retained no fluorescence at low concentrations and very weak fluorescence (below 15%) at higher concentrations. Thus, our panel of PSM does interfere with p-gp to some degree. For liq, all the values were minus zero, suggesting no fluorescence retention, which might correspond to the antagonism of liq in the combinations against Caco-2 cells.

### 3.5. Influence on Gene Expression of MDR Related Genes

In a last set of experiments, we investigated the role of our PSM panel on the expression of genes related to the MDR phenotype. Concentrations corresponded to non-toxic doses, as used in Table 2 and Table 3.

**Figure 5:**
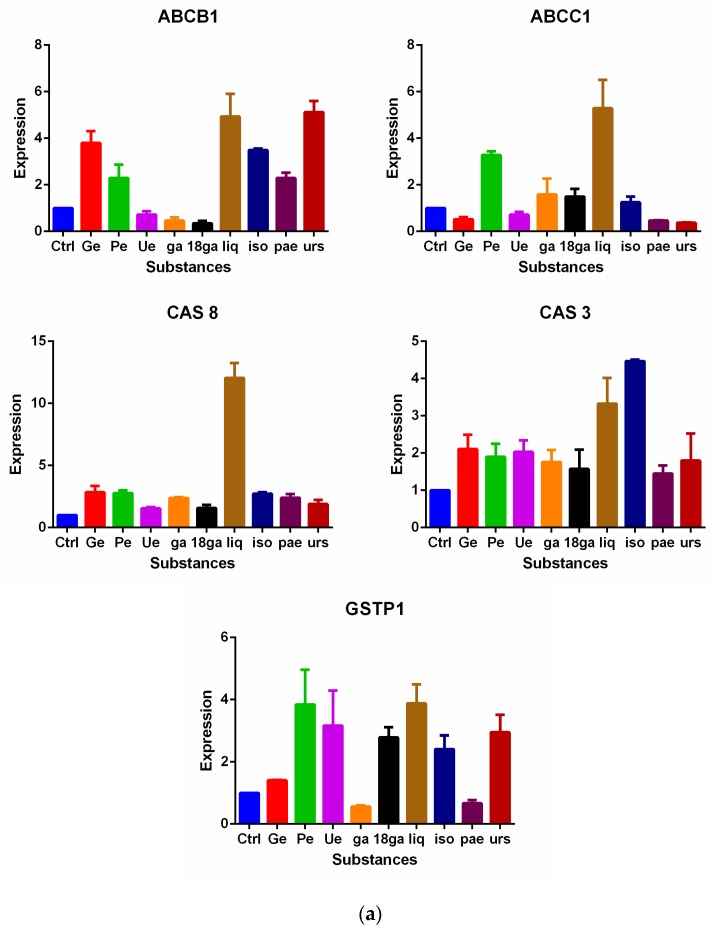

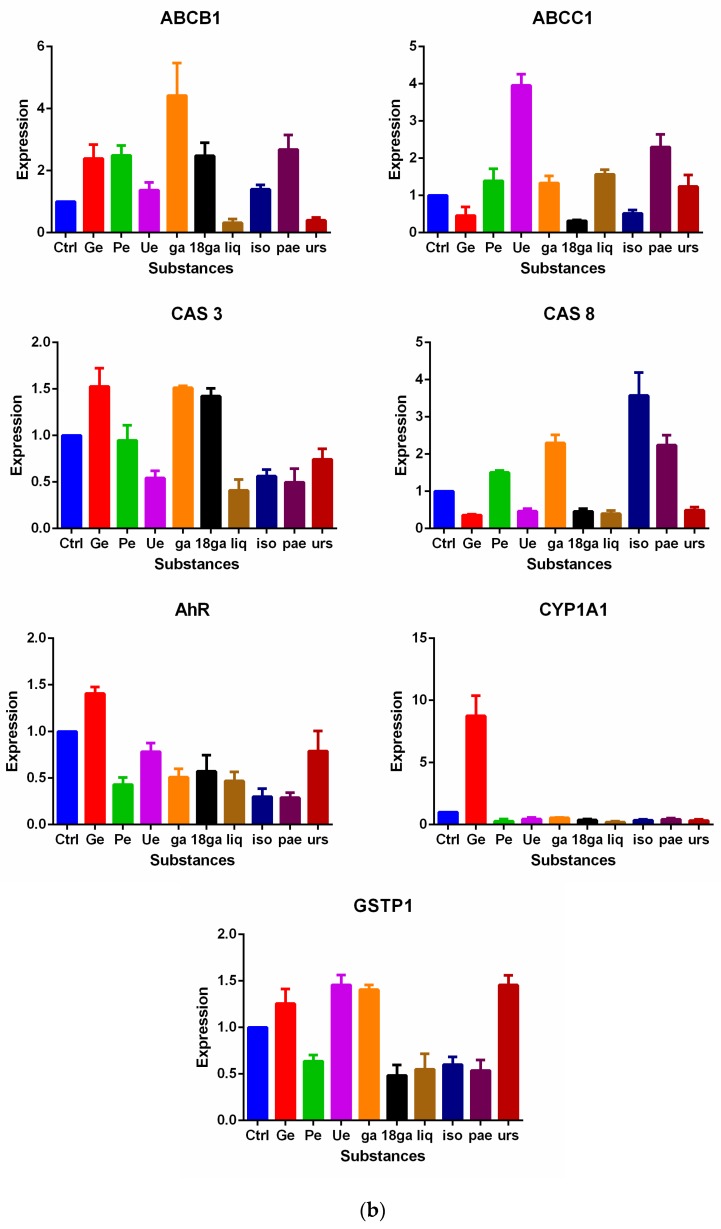
Effects of non-toxic doses of plant secondary metabolite (PSM) on the expression of various genes after 48 h treatment compared to the control (no treatment) in CEM/ADR 5000 cells (**a**) and Caco-2 cells (**b**). Concentrations were the same as used in Table 2 (CEM/ADR 5000 cells) and Table 3 (Caco-2 cells). The results are expressed as the mean ± SD represented at least three independent experiments. The non-treated control group was defined as 1.00.

CEM/ADR 5000 cells and Caco-2 cells were treated with our set of single substances for 48 h, and gene expression of ABCB1 (P-gp), ABCC1 (MRP1), caspase 3 (CAS 3), caspase 8 (CAS 8), glutathione S-transferase pi 1 (GSTP1), AhR, and CYP1A1 were analysed by real-time PCR. Figure 5a shows the expression changes of the candidate genes in CEM/ADR 5000 cells, and Figure 5b in Caco-2 cells.

In CEM/ADR 5000 cells (Figure 5a), Ue, ga, and 18ga reduced ABCB1 expression, whereas the other PSM induced the transporter gene. Ge, Ue, iso, pae, and urs reduced the ABCC1 expression, whereas the other PSM induced its expression. All the substances upregulated the expression of CAS 3 and CAS 8 to some degree. Except for Ga and pae, all PSM activated GSTP1 expression.

In Caco-2 cells (Figure 5b), liq and urs reduced the ABCB1 expression, Ge, 18ga, and iso reduced ABCC1 expression. Ga increased both CAS 3 and CAS 8 expressions, iso and pae stimulated CAS 8 expression. Except for Ge, all the other substances down-regulated AhR and CYP1A1 expression levels. At the same time, Pe, 18ga, ga, iso, and pae reduced the GSTP1 expression whereas the other PSM enhanced it.

## 4. Discussion

The three plant extracts, the root extracts of *Glycyrrhiza glabra*, *Paeonia lactiflora*, and the leaf extract of *Eriobotrya japonica* possess moderate cytotoxic properties which agrees with published data [27,38,58,59,60]. So do the six PSM [30,31,34,35,36,37,38,39,40,44,46,47], which are major PSM in these TCM plants (Table 2). Only ursolic acid showed IC_50_ values below 20 µM. However, the expression of apoptotic marker genes (CAS 3 and CAS 8) was stimulated by the panel, especially in CEM/ADR 5000 cells (Figure 5), which may contribute to the MDR reversal effect seen in two- and three-drug combinations (Table 3 and Table 4).

In this study, all substances showed a chemosensitizing effect for doxorubicin in CEM/ADR 5000 cells and in Caco-2 cells, except for liquiritigenin and isoliquiritigenin in Caco-2 cells. Digitonin further enhanced the effect (Table 3 and Table 4), which might be through the mechanisms explained by Eid et al. [60]. Namely, digitonin may increase the membrane fluidity, increase drug uptake, reduce ABC transporter-mediated drug efflux and induce apoptosis. High reversal ratios were observed for Ue, ga, 18ga, and urs in three-drug combinations in Caco-2 cells (Table 4). Most two- and three-drug combinations produced a synergistic effect (Table 3 and Table 4).

Our hypothesis was that the panel of PSM might interfere with ABC transporters in MDR cancer cells, which overexpress P-gp. In CEM/ADR 5000 cells, most PSM increased calcein or rhodamine 123 retention in a dose-dependent manner to some degree, but none was more active than the positive control verapamil. Furthermore, relatively high concentrations were required to see an effect (Figure 2). As expected, the panel had no effects in sensitive cancer cells, which do not express P-gp (Figure 3). In Caco-2 cells, also relatively high concentrations were required to inhibit P-gp activity (Figure 4). Nevertheless, the data suggest that the PSM might act as inhibitors of ABC transporters. In case of extracts with polyphenols or phenolic PSM, a non-specific inhibition might be due to binding of polyphenols to the transporter proteins by forming hydrogen and ionic bonds [23,61]. However, most PSM of the panel stimulated the expression of ABCB1 or ABCC1 genes (Figure 5), which would also influence drug retention and counteract the inhibitory effects seen in Figure 2 and Figure 4. This effect was not mediated via AhR gene expression (Figure 5b).

The synergistic effects of the PSM panel (Table 2 and Table 3) could also be modulated through influence on metabolic enzymes, such as GST and CYP1A1. Only Ge stimulated the expression of CYP1A1 gene (Figure 5b), where the other compounds reduced its activity. For GSTP1 gene expression, the picture is more complicated. In CEM/ADR 5000 cells, Pe, Ue, 18ga, liq, iso, and urs stimulated the expression of the GSTP1 gene, whereas in Caco-2 cells Ge, Ue, ga, and urs activated this gene, but Pe, 18ga, liq, iso, and pae reduced its expression (Figure 5). We should not forget that CEM/ADR 5000 cells are leukemia cells and Caco-2 colon cells, which might explain the different responses. Furthermore, only non-toxic doses of PSM were applied; higher doses might have caused stronger effects.

Previous studies reported that 18β glycyrrhetinic acid (18ga) inhibited P-gp and MRP1 activity in human MDR cells KB-C2 and KB/MRP [31], showed synergistic effect when combined with doxorubicin or mitomycin in SiHa cells [35], and inhibited P-gp, CYP3A4/5, and depleted glutathione in Caco-2 cells [34]. In our study, 18ga did not inhibit ABC transporters so strongly as the positive control verapamil in both resistant cell lines and only showed synergism in combination with doxorubicin in Caco-2 cells, which might be related to decreased ABCC1, increased Caspase 3, and decreased AhR, CYP1A1, and GSTP1 expressions.

Liquiritigenin and isoliquiritigenin are important components responsible for cytochrome P450 and liquorice interaction [62]. Isoliquiritigenin could chemosensitise human MDR uterine sarcoma cells MES-SA//Dx5-R to doxorubicin by promoting apoptosis and autophagy [38]. In this study, liq was apparently not a relevant MDR modulator in both resistant cell lines (Table 3 and Table 4). Iso chemosensitised MDR cells to doxorubicin partly might through inhibition of ABC transporters, but showed synergism with doxorubicin in CEM/ADR 5000 cells and antagonism in Caco-2 cells (Table 3 and Table 4).

Paeoniflorin modulated MDR against vincristine in human gastric cancer cells SGC7901 partly through inhibition of MDR1, BCL-XL, and BCL-2 expressions [44]. Lipophilic monoterpenes are supposed to target the biomembrane [63]. In our study, paeoniflorin synergistically sensitised CEM/ADR5000 to doxorubicin partly through inhibiting ABC transporters, decreasing ABCC1, increasing caspase expression, and decreasing GSTP1 expressions. Pae did not inhibit ABC transporters in Caco-2 cells and showed additivity in combination, which might be mediated through increasing caspase 8 and reducing AhR, CYP1A1, and GSTP1 expressions.

Ursolic acid (urs) circumvented MDR in human hepatoma cell line R-HepG2 through apoptosis induction but not through P-gp inhibition [46]; ursolic acid increased rhodamine 123 retention in a concentration-dependent manner via inhibiting P-gp in KB-C2 cells [47]. Here, ursolic acid synergistically sensitised both CEM/ADR5000 and Caco-2 cells to doxorubicin by inhibiting ABC transporter activity and expression and interfering with apoptosis- and metabolism-related enzyme expressions. Ursolic acid could be considered as a promising MDR modulator candidate as it showed the highest reversal ratio of our PSM panel in Caco-2 cells.

Natural products can interact in manifold ways with biomembranes, proteins, DNA, RNA, and related enzymes in cells [23,63]. Thus, multitarget effects should be expected when PSM are applied. Our study focused on ABC transporter functions, and gene expressions of ABC transporters, apoptosis and metabolism related enzymes, to illustrate the influence of the three plant extracts and their six SM on MDR. Considering our gene expression data, some results are plausible and would support the MDR reversal activity seen in Table 3 and Table 4. Other results appear contradictory, which may be due to interaction of the PSM in the panel to targets, which were not studied here and the different types of cancer cells employed. To confirm the observed apoptotic activity, further apoptosis experiments need to be conducted, further studies are required to elucidate the exact mechanisms of these substances in MDR and to find out if they are useful in a clinical context.

## 5. Conclusions

In conclusion, extracts of *Glycyrrhiza glabra*, *Paeonia lactiflora* (mixed with *Paeonia veitchii*), and *Eriobotrya japonica*, and their PSM, glycyrrhizic acid, 18β glycyrrhetinic acid, liquiritigenin, isoliquiritigenin, paeoniflorin, and ursolic acid could sensitise MDR cancer cells to doxorubicin in CEM/ADR 5000, and in Caco-2 cells in two- and three-drug combinations. Ue, ga, 18ga, and urs were identified as potential MDR modulator candidates. However, more studies are required to corroborate the findings.

## Figures and Tables

**Table 1:** Primers used in this study for qPCR.

Gene	Sequences	Amplicon
RPL13	Forward: GAGGCGGAACAAGTCCAC	75 nt
	Reverse: AGGATGAGTTTGGAGCGGTA	
ABCB1	Forward: TTACATTCAGGTTTCATTTTGGTG	90 nt
	Reverse: TCCTGTCGCATTATAGCATGA	
ABCC1	Forward: TTGGGATTTTTGCTGTGGA	73 nt
	Reverse: ATATGCCCCGACTTCTTTCC	
GSTP1	Forward: AATGGCAACGGGAAACAGT	77 nt
	Reverse: TTGGTCCTGGAGAAAGGAAG	
CAS 3	Forward: TCTCATGCTGCAGAGGGTACT	77 nt
	Reverse: TGGAAGTTTGAGGTAGCTTCATAG	
CAS 8	Forward: GGAAAGCAATCTGTCCTTCCT	61 nt
	Reverse: CAGCAAATCCAGTCTATTAATTCG	
AhR	Forward: AGAGTTGGACCGTTTGGCTA	86 nt
	Reverse: CGCTGAGCCTAAGAACTGAAA	
CYP1A1	Forward: CCACCAAGAACTGCTTAGCC	74 nt
	Reverse: CAGCTGGGTTTCCAGAGC	

RPL13 is the reference gene. GSTP1 = GSTpi 1; CAS 3 = caspase 3; CAS 8 = caspase 8.

**Table 2:** Cytotoxicity (IC_50_ values *) of individual extracts or secondary metabolites in four kinds of cancer cells.

Sub-stances	CCRF-CEM	CEM/ADR 5000	Caco-2	HCT-116
Dox	0.17 ± 0.06	93.24 ± 19.02	5.40 ± 1.27	0.73 ± 0.04
Dig	10.65 ± 1.07	13.06 ± 2.38	11.28 ± 0.74	0.70 ± 0.10
Ge	96.63 ± 4.21	148.31 ± 9.57	352.97 ± 8.10	305.04 ± 4.78
Pe	56.15 ± 1.99	85.93 ± 3.58	134.64 ± 14.90	202.14 ± 12.09
Ue	50.88 ± 4.51	58.17 ± 3.05	98.95 ± 11.57	67.61 ± 2.89
ga	406.71 ± 24.77	564.83 ± 19.06	602.65 ± 31.39	1415.46 ± 113.43
18ga	76.20 ± 0.93	81.72 ± 0.43	126.18 ± 26.86	113.17 ± 1.77
liq	88.41 ± 4.86	131.61 ± 6.82	746.43 ± 92.93	349.27 ± 16.83
iso	26.09 ± 0.83	55.98 ± 1.60	98.79 ± 11.27	28.26 ± 0.89
pae	802.11 ± 28.93	1376.96 ± 195.12	822.27 ± 297.99	1146.11 ± 120.48
urs	10.28 ± 0.39	14.47 ± 0.65	22.37 ± 1.22	13.10 ± 0.19
Ver	-	130.08 ± 32.49	35.29 ± 3.32	-

* Units for Ge, Pe, and Ue are μg/mL, others are μM. Ge = *Glycyrrhiza glabra* extract; Pe = *Paeonia lactiflora* extract; Ue = *Eriobotrya japonica* extract.

**Table 3:** Cytotoxicity of doxorubicin alone or in combination with a non-toxic concentration of a single substance or with a single substance plus digitonin against CEM/ADR 5000 cells. All data were from at least three independent experiments; they are represented as the mean ± SD. Ge (80 µg/mL—IC_26_): the concentration of Ge in combination was 80 µg/mL and it can cause 26% of cell death in CEM/ADR 5000 cells.

Substances	Two-Drug Combinations (PSM + Dox)				Three-Drug Combinations (PSM + Dox + 0.5 μM Digitonin)			
	IC_50_ (μM of Doxorubicin)	Reversal Ratio	CI	Interpretation	IC_50_ (μM of Dox)	Reversal Ratio	CI	Interpretation
Dox alone	93.24 ± 19.02	1.00	NR	NR	93.24 ± 19.02	1.00	NR	NR
Ge (80 µg/mL—IC_26_)	31.84 ± 5.45	2.93	0.88	+ (slight syn)	23.54 ± 10.76	3.96	0.83	++ (mod syn)
Pe (50 µg/mL—IC_3_)	74.02 ± 9.17	1.26	1.37	-- (mod ant)	59.35 ± 22.22	1.57	1.25	-- (mod ant)
Ue (30 µg/mL—IC_15_)	49.93 ± 13.28	1.87	1.06	± (nearly add)	37.44 ± 7.89	2.49	0.96	± (nearly add)
ga (300 µM—IC_10_)	32.34 ± 2.55	2.88	0.88	+ (slight syn)	26.08 ± 2.18	3.76	0.85	++ (mod syn)
18ga (50 µM—IC_1_)	38.11 ± 6.91	2.45	1.02	± (nearly add)	26.38 ± 10.49	3.53	0.93	± (nearly add)
liq (40 µM—IC_18_)	57.50 ± 8.17	1.62	0.92	± (nearly add)	47.56 ± 7.46	1.96	0.85	++ (mod syn)
iso (20 µM—IC_12_)	31.17 ± 8.60	2.99	0.69	+++ (syn)	25.21 ± 6.10	3.70	0.66	+++ (syn)
pae (500 µM—IC_11_)	40.27 ± 5.13	2.32	0.79	++ (mod syn)	34.17 ± 9.04	2.73	0.76	++ (mod syn)
urs (6 µM—IC_17_)	30.13 ± 10.49	3.10	0.74	++ (mod syn)	25.96 ± 7.67	3.59	0.73	++ (mod syn)

NR: not relevant. CI: combination index.

**Table 4:** Cytotoxicity of doxorubicin alone or in combination with a non-toxic concentration of a single substance or with a single substance plus digitonin against Caco-2 cells. All data were from at least three independent experiments and represented as the mean ± SD.

Substances	Two-Drug Combinations (PSM + Dox)				Three-Drug Combinations (PSM + Dox + 0.5 μM Digitonin)			
	IC_50_ (μM of Dox)	Reversal Ratio	CI	Interpretation	IC_50_ (μM of Dox)	Reversal Ratio	CI	Interpretation
Dox. alone	5.40 ± 1.27	1.00	NR	NR	5.40 ± 1.27	1.00	NR	NR
Ge (200 µg/mL—IC_5_)	3.21 ± 0.72	1.68	1.16	- (slight ant)	2.14 ± 1.08	2.52	1.01	± (nearly add)
Pe (25 µg/mL—IC_10_)	3.44 ± 0.97	1.32	0.82	++ (mod syn)	0.42 ± 0.20	2.52	0.31	+++ (syn)
Ue (40 µg/mL—IC_6_)	1.10 ± 0.08	4.91	0.60	+++ (syn)	0.52 ± 0.18	10.38	0.53	+++ (syn)
ga (350 µM—IC_10_)	1.74 ± 0.56	3.10	0.90	± (nearly add)	0.68 ± 0.27	7.94	0.75	++ (mod syn)
18ga (25 µM—IC_20_)	2.08 ± 0.97	2.60	0.58	+++ (syn)	0.96 ± 0.45	5.63	0.42	+++ (syn)
liq (200 µM—IC_20_)	11.45 ± 3.78	0.47	2.39	--- (ant)	7.83 ± 3.65	0.69	1.76	--- (ant)
iso (30 µM—IC_23_)	6.36 ± 1.78	0.85	1.48	--- (ant)	4.61 ± 1.29	1.17	1.20	-- (mod ant)
pae (200 µM—IC_27_)	3.59 ± 0.79	1.50	0.90	± (nearly add)	3.02 ± 0.89	1.79	0.84	++ (mod syn)
urs (8 µM—IC_12_)	1.45 ± 0.55	3.72	0.63	+++ (syn)	0.32 ± 0.07	16.88	0.46	+++ (syn)

NR: not relevant. CI: combination index.
